# Serum levels of Th17-related cytokines in Behcet disease patients after cataract surgery

**Published:** 2011-05-31

**Authors:** Shuhong Jiang, Xialin Liu, Lixia Luo, Bo Qu, Xiangkun Huang, Ying Lin, Shaobi Ye, Yizhi Liu

**Affiliations:** 1 State Key Laboratory of Ophthalmology, Zhongshan Ophthalmic Center, Sun Yat-sen University, Guangzhou, China; 2 People’s Hospital of Inner Mongolia, Hohhot, China

## Abstract

**Purpose:**

To investigate the profile of T-helper type 17 (Th17) cell–related cytokines (interleukin-23 [IL-23], IL-27, IL-17 and interferon-γ [IFN-γ]) in postoperative inflammation in patients with Behcet disease (BD) after cataract surgery.

**Methods:**

Serum was collected from seven BD patients with complicated cataract, and from nine controls with uncomplicated cataract, before cataract surgery, and again 1, 7, 30, and 90 days after surgery. In addition, aqueous humor was collected at commencement of surgery. The protein levels of IL-23, IL-27, IL-17, and IFN-γ in the serum and in the aqueous humor were measured by an enzyme-linked immunosorbent assay. A laser flare-cell photometer was used to quantify intraocular inflammation.

**Results:**

Serum IL-23, IL-27, and IFN-γ levels were significantly increased after cataract surgery in the BD versus the control patients. In the BD patients, serum levels of IFN-γ and IL-27 correlated strongly with aqueous flare values and cell counts. Remarkably, the levels of serum IL-27 were significantly associated with serum IFN-γ levels in BD patients (r=0.796; p=0.002).

**Conclusions:**

Our data indicates that serum IFN-γ and IL-27 levels are significantly elevated in BD versus control patients and are strongly associated with post-operative intraocular inflammation.

## Introduction

Behcet disease (BD) is a chronic multisystem disorder characterized by recurrent uveitis, oral aphthae, genital ulcers, and skin lesions. Behcet uveitis is one of the most common types of uveitis occurring in China [1]. Cataract formation, the most frequent anterior segment complication of uveitis in BD patients, is a major cause of decreased visual acuity [2]. Treatment of these cataracts is not easy because surgical procedures can provoke inflammation. Suppression of the inflammatory response is critical for the success of surgery in these patients.

Although the pathogenesis of BD is still unclear, several reports suggest that an autoimmune response may play an important role in the development of inflammation in these patients [3]. Previous studies have suggested that interferon-γ (IFN-γ) are present in BD patients [4,5]. Recent studies have shown increased levels of other T-helper type 17 (Th17) cell associated cytokines, such as interleukin 23 (IL-23) and IL-17, in BD patients with active uveitis [6]. In addition, previous studies have shown that the presence of IL-27 may limit Th17 mediated uveitis [7]. However, the association of these cytokines with postoperative intraocular inflammatory activity in BD patients is not yet clear. In this study, we determine the correlation between the serum concentrations of these cytokines in BD patients and the intraocular variables of the disease activity.

## Methods

### Patients

Seven eyes with complicated cataracts, from seven BD patients, were included in the study. BD disease was diagnosed according to the criteria determined by the International Study Group for BD disease [1,8]. Cataract surgery was performed on these patients after at least 3 months of inactive uveitis. These patients had received prednisone at a low dose (<20 mg/d), but no other immunosuppressive agents, for at least 2 months before the first sampling. Nine eyes of nine uncomplicated cataract patients (age related cataract patients, n=6; congenital cataract patients, n=3) were selected as controls, were matched for age and sex and the surgery was performed on the same day as the BD patients. None of these controls had suffered previous ophthalmic disease or received any medication known to influence cataract formation. Patients and controls underwent phacoemulsification between July 2008 and March 2009 at Zhongshan Ophthalmic Center (Guangzhou, China). This study was performed in accordance with the Declaration of Helsinki and with the approval of the local ethical committee. Informed consent was obtained from all patients and controls.

### Post-operative management

Standard cataract procedure was performed by the same experienced cataract surgeon (Yizhi Liu) on all patients as described [9,10]. All patients received dexamethasone-tobramycin eye drops four times daily during the first week, and twice a day during the second week, after which treatment was discontinued except when signs of severe postoperative inflammation were present. Patients with BD disease also received prednisolone (initial dose, 1 mg/kg), which was gradually reduced, based on the extent of intraocular inflammation.

### Measurement of anterior intraocular inflammation

Aqueous flare measurements and cell counts were made using a slit lamp and laser flare-cell photometer FC-2000 (Kowa, Tokyo, Japan) as described in previous studies [9-11]. Measurements of flare intensity and cell count were done before surgery with follow-up measurements 1, 7, 30, and 90 days after surgery. Three individual measurements from each eye were averaged; measurements affected by artifacts were discarded. Flare and cell readings were expressed as photon counts per millisecond, and cells per 0.5 mm^3^, respectively.

### Sample collection

Blood samples (2 ml) were collected before surgery and 1, 7, 30, and 90 days after surgery. Samples of aqueous humor (AqH; 50–100 µl) were collected from the paracentesis site for phacoemulsification. After centrifugation at 300× g for 5 min, serum and AqH samples were separated into cellular components and supernatant and were frozen at −80 °C until use.

### Enzyme-linked immunosorbent assay

The concentrations of IL-23, IL-27, IL-17, and IFN-γ in sera or AqH were measured with commercial sandwich enzyme-linked immunosorbent assays, following the manufacturer’s instructions (DuoSet ELISA Development System; R&D Systems, Minneapolis, MN). The limit of detection was 15 pg/ml.

### Statistical analysis

The data were analyzed using SPSS 10.0 for Windows XP (SPSS Science, Chicago, IL). All data were expressed as mean±SD. Variables at the follow-up times were compared using Student’s *t* test. Linear regression was used to assess the influence of potential risk factors (age, sex, “phaco-time” and “phaco-energy”) on flare values and cell counts. Correlation coefficients were calculated with the Spearman correlation coefficient test. A p≤0.05 was considered statistically significant.

## Results

### General data of the BD patients and control subjects

Patient demographics and clinical features are summarized in Table 1. Patient ages ranged from 15 to 52 years in the BD disease group and from 17 to 60 years in the control group. Preoperative and postoperative courses were uneventful in all patients. Other variables (such as age, sex, cataract type, phaco-time, phaco-energy and phaco-time × phaco-energy) were not correlated with flare values, cell counts, and cytokine levels in the serum and the AqH.

**Table 1:** Demographic and clinical features of BD patients and control subjects.

**Parameters**	**Patients with BD disease**		**Control subjects**	
n	7		9	
Age (y)	36.71±13.78		40.43±11.06	
Sex (male/female)	3/4		4/5	
Phaco-time (s)	24.43.±16.17		28.15±19.26	
Phaco-energy (%)	11.29±3.52		12.37±2.48	
Intraocular inflammation	Flare value	Cell counts	Flare value	Cell counts
Preoperative	6.07±2.57	0.57±0.34	1.71±1.46	0.22±0.17
Postoperative				
1 day	72.71±20.36***	34.77±9.57***	23.21±8.34***	12.40±2.57***
7 d	52.94±14.68***	20.12±6.81***	12.96±4.63***	5.98±2.34***
30 d	12.52±3.51**	2.74±1.40**	2.21±1.45	0.61±0.53*
90 d	5.88±2.30	0.70±0.64	1.81±1.37	0.28±0.19

*p<0.05; **p<0.01; ***p<0.001 (compared with preoperative levels).

### Cytokine levels of BD patients and controls before surgery

**Figure 1:**
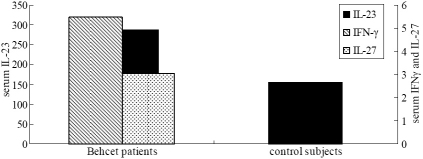
The cytokine levels (pg/ml) of BD patients and controls before surgery.

Before surgery, IL-17 was undetectable in the serum of all BD patients and control subjects. IL-23 was expressed in serum of both BD patients and control subjects. The low concentrations of IL-27 and IFN-γ were detected in serum of BD patients. However, IL-27 and IFN-γ were lower than the detection level in control subjects (Figure 1). AqH samples were collected at the initiation of cataract surgery, and protein levels of the Th17-associated cytokines were measured. The results showed that IL-23, IL-27, IL-17, and IFN-γ were undetectable in all AqH samples of both BD patients and controls.

### The change in cytokine levels of BD patients following surgery

**Figure 2:**
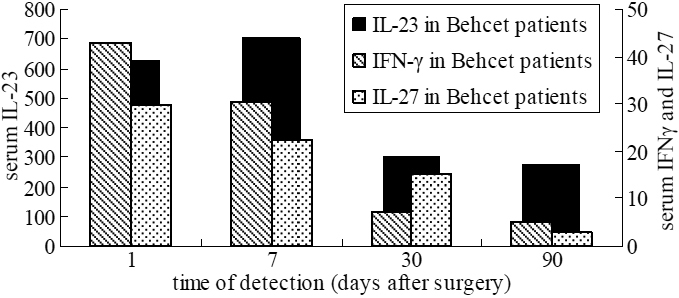
The change in cytokine levels (pg/ml) of BD patients after surgery.

In BD patients, serum IL-23, IFN-γ, and IL-27 levels significantly increased and peaked on day 1 post-surgery. Then IFN-γ and IL-27 levels decreased rapidly during the first week, although IL-23 level still elevated. All these cytokines gradually returned to preoperative levels by 90 days after surgery (Figure 2). IL-17 was undetectable in serum of BD patients after surgery.

### The change in cytokine levels of controls following surgery

**Figure 3:**
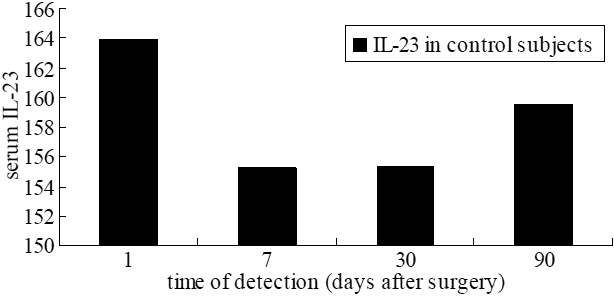
The change in cytokine levels (pg/ml) of controls after surgery.

In control subjects, serum IL-23 levels increased on day 1 post-surgery, decreased during the first month, and gently elevated on day 90 post-surgery. IL-17, IFN-γ, and IL-27 were still lower than the detection level after surgery (Figure 3).

### Comparison of cytokine levels between BD patients and controls

**Figure 4:**
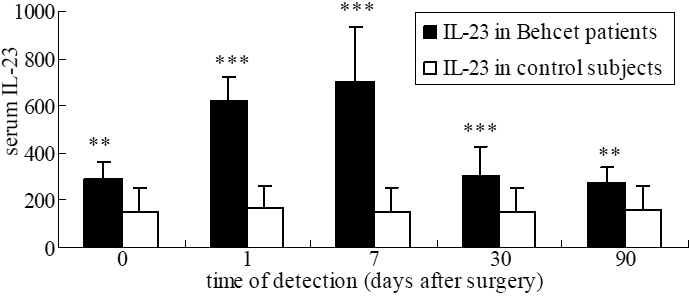
The comparison of cytokine levels (pg/ml) between BD patients and controls.

Serum IL-23 levels were considerably higher in BD patients compared to control subjects preoperatively and on days 1, 7, 30, and 90 days postoperatively (p=0.003, p<0.001, p<0.001, p<0.001, and p=0.007, respectively; Figure 4).

### Significant correlation between serum levels of Th17 cytokines and inflammatory variables in BD patients

**Figure 5:**
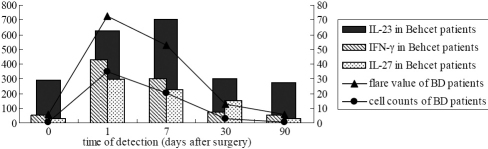
The correlation between serum levels of Th17 cytokines (pg/ml) and inflammatory variables(flare value: pc/ms; cell counts: cells/0.5mm^3^) in BD patients.

The results showed that, for both groups, the aqueous flare value and cell count increased after surgery and achieved the highest level on day 1 post-surgery, and then decreased rapidly during the first week, which was then followed by a more gradual reduction. Serum IFN-γ and IL-27 levels correlated strongly with aqueous flare value and cell counts in BD patients, but that serum IL-23 levels did not (Table 2 and Figure 5). Of note, serum IL-27 levels in BD patients were significantly associated with serum IFN-γ levels (r=0.796; p=0.002). No correlation has been found between serum IL-23 levels and postoperative inflammation, although serum IL-23 levels were elevated after surgery in control subjects.

**Table 2:** Correlation analysis of serum cytokine levels and inflammatory variables in BD patients.

** **	**Serum IL-23**		**Serum IFN-γ**		**Serum IL-27**	
**Variables**	**r**	**p**	**r**	**p**	**r**	**p**
Flare value	0.214	0.107	0.764	0.013	0.755	0.026
Cell counts	0.549	0.093	0.803	0.002	0.694	0.011

All correlations were calculated with the Spearman correlation coefficient test.

## Discussion

Cataract, resulting from chronic intraocular inflammation or from the corticosteroid therapy given for inflammation, is a common complication of uveitis in BD patients. In both children and adults, the importance of preoperative and postoperative inflammation control is paramount [12,13]. The Th17 cell is a unique CD4-positive (CD4+)T-cell subset characterized by the production of IL-17. Recent data from humans and mice suggests that Th17 cells play an important role in the pathogenesis of a diverse group of immune-mediated diseases, including BD and Vogt-Koyanagi-Harada (VKH) disease [6,9,14,15]. It has been shown that BD patients with active uveitis exhibit higher IL-17 and IL-23 levels as compared with patients with inactive uveitis, or normal controls [6].

IL-23, a member of the IL-12 family of cytokines, plays an important role in Th17 maintenance [16]. In the present study, we measured the dynamic changes of serum IL-23 in BD patients and control subjects both before and after surgery. The results showed that serum IL-23 was upregulated in BD patients, compared with controls, both before and after surgery. However, no statistical association was found between the increased serum IL-23 levels and intraocular inflammation in BD patients. The exact role of IL-23 on postoperative uveitis is not well understood and further studies are needed to define this in more detail.

Previous studies found that an extremely Th1-rich cytokine environment was formed in Behçet uveitis [4,17]. The present study was also done to evaluate the influence of cataract surgery on IFN-γ expression. Our results showed significantly upregulated serum levels of IFN-γ after surgery in BD patients which correlated with inflammatory variables of postoperative uveitis. These results are consistent with those of previous studies that documented that BD patients with active uveitis showed increased levels of IFN-γ in both the serum and the AqH as compared to normal controls [4-6,14]. Unexpectedly, it has been shown that almost half of all the Th17 cells co-express IFN-γ in humans, and that IFN-γ may inhibit IL-17 expression [6,16,18]. The exact relationship between IL-17 and IFN-γ in postoperative uveitis is not well understood and further studies are needed to describe this in more detail.

The immunologic role of IL-27 has been documented and discussed in relation to various Th1/Th17-mediated inflammatory diseases [19,20]. However, its importance in postoperative uveitis is unknown. In this study, we invested the variation in the levels of serum IL-27 before and after surgery. The results showed that serum IL-27 levels in BD patients were significantly elevated after surgery and correlated significantly with disease severity and serum IFN-γ levels. These findings are consistent with earlier reports about the cytokine environment in psoriasis [21]. In addition, it has been shown that IL-27, as constitutively expressed in retinal ganglion and photoreceptor cells, is upregulated by IFN-γ, and inhibited Th17 proliferation in uveitis [7]. Thus, the results of our study indicate that elevated IL-27 in the sera of BD patents after surgery plays an important role in postoperative inflammation. Further studies are needed to assess whether IL-27 plays a protective or pathogenic role in postoperative uveitis in BD patients.

Although IL-17 was undetectable in the serum of all subjects in the present study, a previous study found that IL-17 production by CD4^+^ T cells increased significantly in the presence of IL-23 in BD patients with active uveitis [6]. In addition, it was reported that IL-17 is present in active uveitic eyes in BD patients [4]. However, in our study, IL-23, IL-17, IL-27, and IFN-γ were undetectable in the AqH of both BD patients and controls. These results might be attributed to the inactive stages of inflammation in BD patients, to the use of topical corticosteroids and to the different assays used for study.

Our study showed that the serum levels of IL-23, IFN-γ, and IL-27 were significantly elevated after cataract surgery in BD patients. These findings support previous reports about the effects of cataract surgery on age-related cataract patients and patients with VKH disease [9,22]. A significant increase of serum levels of cytokines in cataract patients following cataract surgery, have been described in those studies. In addition, serum cytokines relating to the activity of the inflammatory response in BD patients were most prominent in several studies [23,24]. As memory T cells can be potent inducers of the cytokines tested, the increased serum levels of IL-23, IFN-γ, and IL-27 following surgery strongly suggest an underlying inflammation and immune hypersensitivity reaction in patients with BD disease that is provoked by the cataract trauma. These findings may be associated with several factors: a more severe postoperative disturbance of the blood-aqueous barrier, a greater uveal reaction to surgical trauma and some systemic response triggered in the circulation. Further studies are needed to assess the real role of these cytokines in postoperative uveitis in BD patients.

In conclusion, this study showed that increased serum levels of Th17-related cytokines, particularly of IL-27 and IFN-γ, are possibly involved in the development of postoperative inflammation in BD patients. However, given the meticulous cross-regulatory mechanisms in Th17 cytokines, further study is needed to investigate their detailed role in the postoperative ocular inflammation of different aspects of uveitic diseases.
